# Anesthetic Precision in Severe Hypertrophic Cardiomyopathy: Navigating Perioperative Challenges

**DOI:** 10.7759/cureus.72947

**Published:** 2024-11-03

**Authors:** Poonam Hannurkar, Urmila Phad, Chhaya Suryawanshi

**Affiliations:** 1 Department of Anesthesiology, Dr. D. Y. Patil Medical College, Hospital & Research Centre, Dr. D. Y. Patil Vidyapeeth (Deemed to be University), Pune, IND

**Keywords:** atrial fibrillation, ileal adenocarcinoma, left ventricular outflow tract obstruction, severe hypertrophic cardiomyopathy, vasopressors

## Abstract

Managing a patient with hypertrophic obstructive cardiomyopathy (HOCM) presents a significant challenge for any anesthesiologist. A deep understanding of the pathophysiology, alongside meticulous planning and hemodynamic monitoring, is essential. Key considerations include maintaining sinus rhythm, ensuring adequate preload and afterload, and preventing tachycardia. Here, we present a unique case of ileal adenocarcinoma scheduled for resection and anastomosis in a patient with a 40-year history of seizure disorder and a severe left ventricular outflow tract gradient of 110 mm Hg, illustrating how a vigilant and methodical approach can lead to successful outcomes in such complex cases.

## Introduction

Hypertrophic cardiomyopathy (HCM) is a rare autosomal dominant genetic disorder affecting the myocardium, with a prevalence of approximately one in 500 adults [1]. It can be classified into asymmetrical, concentric, midventricular, and apical forms. In 70% of cases, HCM involves left ventricular outflow tract (LVOT) obstruction. Patients may be asymptomatic or present with atrial fibrillation, atrial flutter, angina pectoris, heart failure, or sudden cardiac death [2]. It is also a leading cause of sudden cardiac death in younger individuals [3]. Perioperative management in these cases focuses on preventing exacerbation of LVOT obstruction [4]. This case presentation underscores that high-risk patients can be successfully managed through thorough preoperative evaluation and vigilant, meticulous intraoperative hemodynamic monitoring.

## Case presentation

Herein, we present an intriguing and complex case of a 77-year-old female patient weighing 44 kg, diagnosed with adenocarcinoma of the ileum, associated hypertrophic obstructive cardiomyopathy (HOCM), and a seizure disorder. She was scheduled for resection and anastomosis with ileostomy. The patient’s history revealed a diagnosis of generalized tonic-clonic seizures 40 years prior, for which she was on phenobarbital 60 mg twice daily and levetiracetam 500 mg three times daily. Four years ago, she experienced episodes of palpitations, chest pain, and syncope, leading to a diagnosis of HCM. She was initiated on metoprolol at a dose of 40 mg twice daily.

**Figure 1:**
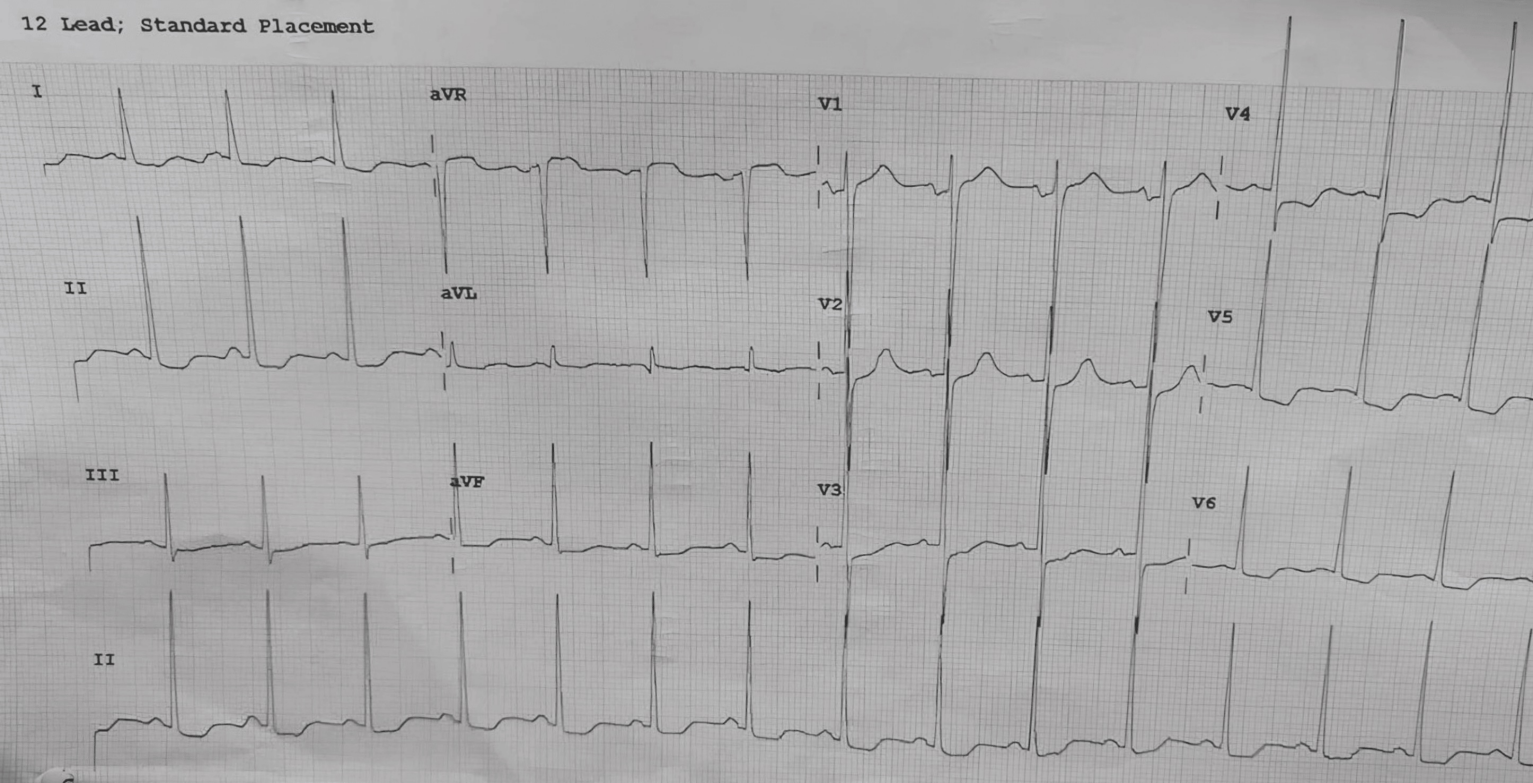
ECG illustrating LVH LVH, left ventricular hypertrophy

Eight months prior, the patient developed atrial fibrillation, prompting the addition of amiodarone twice daily and warfarin. She improved symptomatically and returned to sinus rhythm. Examination revealed a pulse rate of 76 beats per minute, a blood pressure of 130/70 mm Hg, and an oxygen saturation of 97% on room air. The airway and systemic examinations were normal, except for an apical systolic murmur. All laboratory tests were normal, except for the international normalized ratio, which was 2.5. The ECG indicated left ventricular hypertrophy (Figure 1).

**Figure 2:**
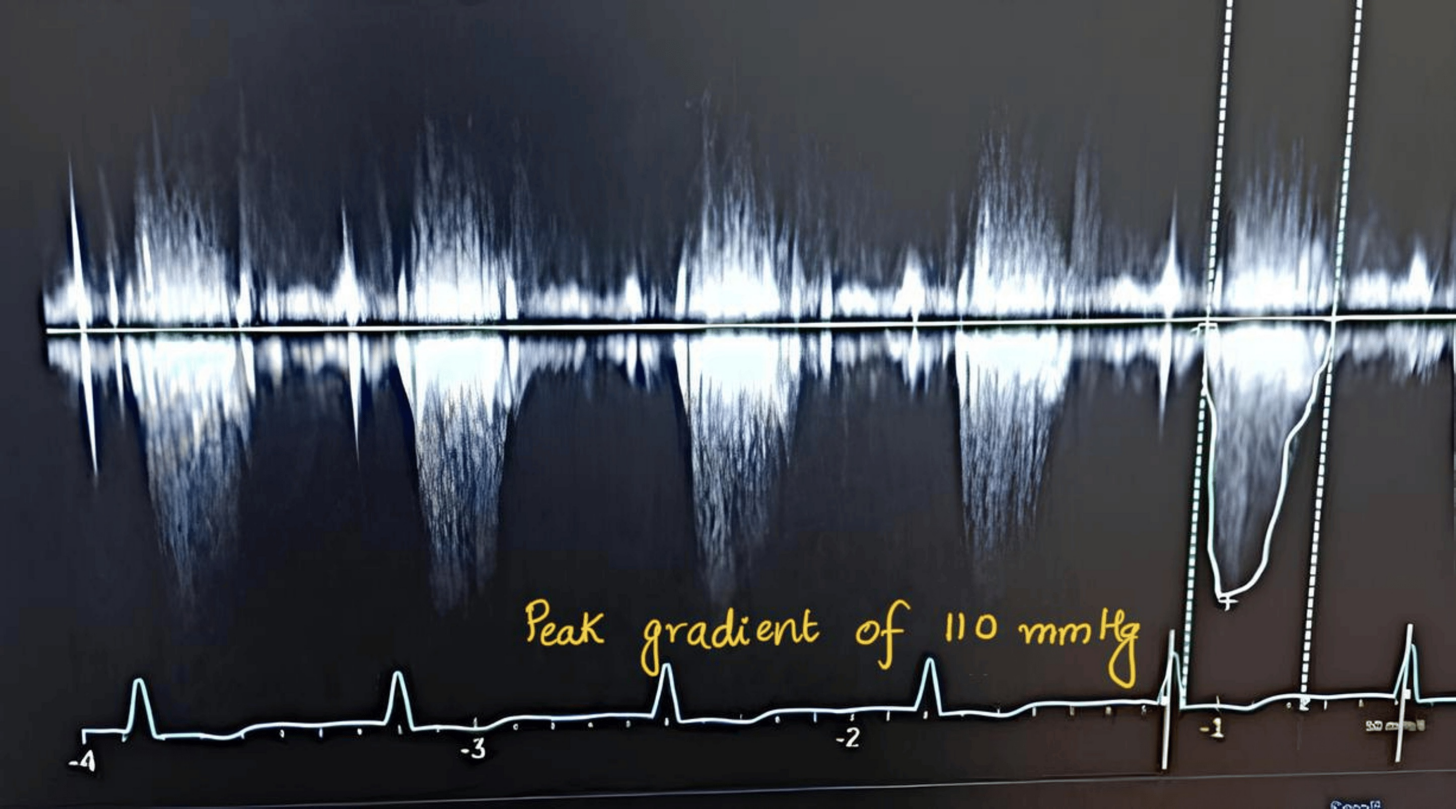
Doppler image displaying LVOT gradient LVOT, left ventricular outflow tract

**Figure 3:**
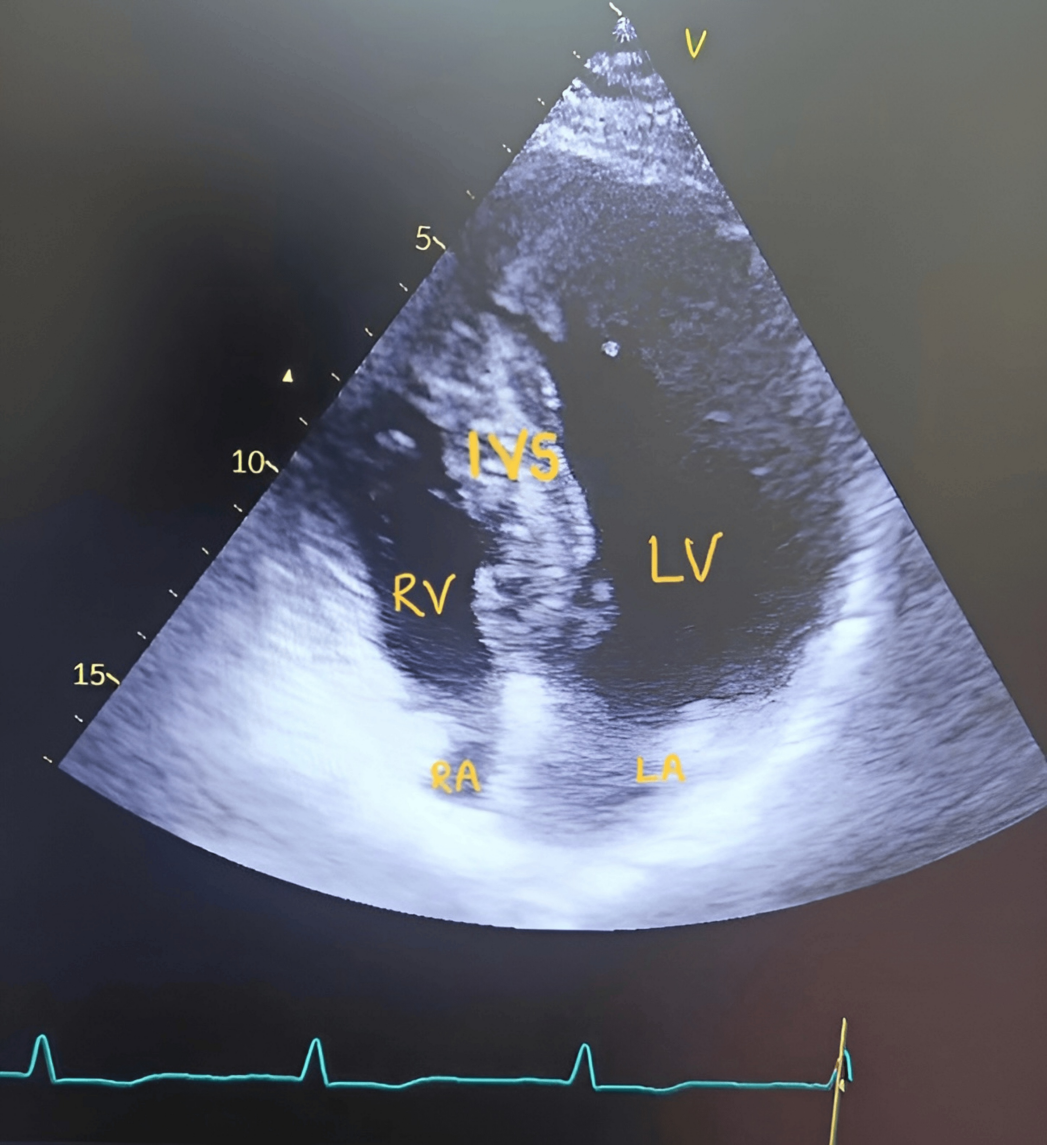
2D echocardiography illustrating hypertrophy of the IVS and LV IVS, interventricular septum; LA, left atrium; LV, left ventricle; RA, right atrium; RV, right ventricle

Echocardiography revealed HOCM with asymmetrical septal hypertrophy, grade 2 mitral regurgitation, a mildly dilated left atrium, and a severe LVOT gradient of 110 mm Hg (Table 1, Figure 2, Figure 3).

**Table 1:** Findings from 2D echocardiography and color Doppler imaging IAS, interatrial septum; IVS, interventricular septum; LVOT, left ventricular outflow tract

Parameter	Findings
Left atrium	Mildly dilated
Left ventricle	Hypertrophic
Right atrium and ventricle	Normal size
IVS	Intact (18 mm), asymmetrical septal hypertrophy
IAS	Intact
Ejection fraction	60%
Pulmonary pressure gradient	5 mm Hg
Aortic pressure gradient	4 mm Hg
Mitral valve	Grade 2 regurgitation
LVOT gradient	110 mm Hg
Tricuspid valve	Mild regurgitation

All cardiac medications and antiepileptic drugs were administered up to the day of surgery, and warfarin was bridged with enoxaparin 40 mg twice daily, with the last dose taken 12 hours prior to the procedure. In the operating theater, standard ASA monitors (including ECG with approximated ST segment analysis) were attached. Additionally, external defibrillator pads were applied, as patients with HOCM are prone to dysrhythmias.

The right radial artery was cannulated under local anesthesia for beat-to-beat blood pressure monitoring and goal-directed fluid therapy. An 18-gauge wide bore cannula was secured in the left hand, and central venous cannulation of the right internal jugular vein was performed. A prophylactic antibiotic injection of cefuroxime 1.5 grams was administered 30 minutes before the incision. An epidural catheter was inserted at the T10-11 space and secured 10 cm prior to induction. The patient was preloaded with 500 milliliters of normal saline and premedicated with midazolam 1 mg and fentanyl 2 µg/kg. After adequate preoxygenation, induction was achieved with etomidate 0.3 mg/kg, and vecuronium 0.1 mg/kg was used for endotracheal intubation. Lignocaine 60 mg intravenously was administered to attenuate the stress response.

Maintenance of anesthesia was carried out with 50% nitrous oxide in oxygen, sevoflurane (MAC between 0.8% and 1%), and boluses of vecuronium. Antiarrhythmic medications such as amiodarone and emergency drugs including atropine, adrenaline, noradrenaline, phenylephrine, vasopressin, and a defibrillator were kept readily available for any inadvertent arrhythmic events. Immediately following induction, the patient experienced hypotension of 80/50 mm Hg, which was managed with a bolus of 100 micrograms of phenylephrine to maintain systemic vascular resistance (SVR).

Pulse pressure variation was utilized for fluid management, and central venous pressure was maintained between 10 and 13 cm H2O throughout the surgery. Intraoperative analgesia was sustained with an epidural infusion of 0.2% ropivacaine at 5 ml/hr, along with an injection of paracetamol 1 gram. Hemodynamically, the intraoperative heart rate fluctuated between 60 and 86 beats per minute, while systolic blood pressure ranged from 95 to 135 mm Hg. The surgery lasted for six hours, after which the patient was successfully reversed with sugammadex and extubated.

Post-extubation pain was managed with an epidural infusion of ropivacaine 0.125% at 4 ml/hr for 48 hours to prevent sympathetic stimulation and tachycardia. The patient was transferred to the ICU for postoperative care, where she was monitored for two days before being moved to the ward. Her postoperative stay was uneventful, and she was discharged on day 7. Follow-up appointments were conducted at two weeks and again at four weeks in the surgical outpatient department, during which she resumed her routine daily activities.

## Discussion

The majority of patients with HCM exhibit LVOT obstruction either at rest or under stress, including during anesthesia. This obstruction arises from the systolic anterior motion of the mitral valve, leading to contact between the mitral valve and the ventricular septum, which can result in mild to moderate mitral regurgitation [5]. The clinical progression of the disease typically begins with outflow obstruction and can progress to diastolic dysfunction, impaired coronary vasodilator reserve, myocardial ischemia, and various arrhythmias, including supraventricular and ventricular tachyarrhythmias [6].

In our patient, medical management included metoprolol, amiodarone, and warfarin. Beta-blockers help reduce heart rate at rest and during exertion, which in turn prolongs diastolic filling time, decreases myocardial oxygen demand, and improves overall exercise tolerance [7]. Amiodarone provides long-term arrhythmia control and enhances survival [8]. Warfarin was initiated to mitigate the risk of thromboembolism, particularly given the patient’s dilated left atrium and arrhythmias such as atrial fibrillation and flutter [9].

Anesthetic induction in patients with HCM presents unique challenges. Preoperatively, anesthesiologists must assess the severity of LVOT obstruction, signs of heart failure, the type of surgery, and expected blood loss, while also considering any additional risk factors.

In this case, the LVOT gradient was measured at 110 mm Hg at rest, indicating severe obstruction, along with grade 2 mitral regurgitation and a mildly dilated left atrium; however, no signs of heart failure were observed. Additionally, the patient had a seizure disorder and had been on a regular regimen of phenobarbital (60 mg) for 40 years.

The patient was diagnosed with adenocarcinoma of the ileum, for which resection and anastomosis with ileostomy were planned, constituting a time-sensitive surgical intervention. To maintain adequate preload and prevent exacerbation of LVOT obstruction, it was essential to avoid prolonged preoperative fasting and dehydration. Securing a wide-bore cannula for rapid fluid resuscitation was also crucial.

The intraoperative management goals for noncardiac surgery under general anesthesia in such cases focus on preventing increases in LVOT obstruction, diastolic dysfunction, and arrhythmias. To achieve these objectives, it is important to prioritize maintaining SVR, optimizing left ventricular filling, preserving sinus rhythm, and reducing both heart rate (chronotropy) and contractility (inotropy).

Preloading and premedication are critical, as they help mitigate the effects of positive pressure ventilation and the depressant effects of induction agents [10]. Extreme caution is required during induction, laryngoscopy, intubation, positioning, and extubation, as tachycardia is undesirable and can compromise ventricular filling.

Maintaining fluid balance during the intraoperative period is complex. Optimal fluid therapy is necessary, as both hypotension and fluid overload can have serious consequences [11]. In this case, blood loss was minimal at approximately 300 ml, which was replaced with crystalloids. If hypotension occurs during surgery, vasopressors that increase afterload without enhancing contractility should be administered. Phenylephrine is preferred due to its ability to increase SVR and induce reflex bradycardia, both of which help maintain LVOT patency [12].

High tidal volume and positive end-expiratory pressure should be avoided in these patients, as they can reduce preload and exacerbate LVOT obstruction. Therefore, it is advisable to use smaller tidal volumes with higher respiratory rates to ensure adequate minute ventilation [13].

Intraoperative vigilance to prevent sympathetic stimulation, tachycardia, and hypotension is paramount. The successful outcome of surgery in our case can be attributed to goal-directed fluid therapy and prompt management to maintain stable intraoperative hemodynamics.

## Conclusions

Patients with positive genetic markers for HOCM should undergo a comprehensive preoperative cardiac evaluation. Even individuals without apparent signs or symptoms of outflow obstruction may experience dynamic obstruction during general anesthesia. Multimodal monitoring, goal-directed fluid therapy, and vigilant hemodynamic management are essential to preventing complications such as arrhythmias and hypotension.

This case underscores the importance of individualized anesthetic strategies, which include the application of external defibrillator pads, precise fluid balance management, and careful medication use to avoid exacerbating LVOT obstruction. Cautious perioperative care and attentive intraoperative monitoring are key factors contributing to successful surgical outcomes in patients with HOCM.

## References

[1] Wheeler MT, Ashley EA (2015). Hypertrophic cardiomyopathy: can the horse be put back in the barn?. J Am Coll Cardiol.

[2] Ibrahim IR, Sharma V (2017). Cardiomyopathy and anaesthesia. BJA Educ.

[3] Weissler-Snir A, Allan K, Cunningham K (2019). Hypertrophic cardiomyopathy-related sudden cardiac death in young people in Ontario. Circulation.

[4] Gajewski M, Hillel Z (2012). Anesthesia management of patients with hypertrophic obstructive cardiomyopathy. Prog Cardiovasc Dis.

[5] Değirmenci H, TAŞ HG (2022). Tips in anesthetic techniques in hypertrophic cardiomyopathy. Cardiol Res Rep.

[6] Poliac LC, Barron ME, Maron BJ (2006). Hypertrophic cardiomyopathy. Anesthesiology.

[7] Taha M, Dahat P, Toriola S (2023). Metoprolol or verapamil in the management of patients with hypertrophic cardiomyopathy: a systematic review. Cureus.

[8] McKenna WJ, Harris L, Rowland E (1984). Amiodarone for long-term management of patients with hypertrophic cardiomyopathy. Am J Cardiol.

[9] Nasser MF, Gandhi S, Siegel RJ, Rader F (2021). Anticoagulation for stroke prevention in patients with hypertrophic cardiomyopathy and atrial fibrillation: a review. Heart Rhythm.

[10] Nama RK, Parikh GP, Patel HR (2015). Anesthetic management of a patient with hypertrophic cardiomyopathy with atrial flutter posted for percutaneous nephrolithotomy. Anesth Essays Res.

[11] Sahoo RK, Dash SK, Raut PS, Badole UR, Upasani CB (2010). Perioperative anesthetic management of patients with hypertrophic cardiomyopathy for noncardiac surgery: a case series. Ann Card Anaesth.

[12] Mithani M, Flatow G, Chyfetz MA (2021). Management of a critically ill patient with severe hypertrophic obstructive cardiomyopathy presenting for emergent craniotomy due to subdural hemorrhage. Cureus.

[13] Moreno Garijo J, Ibáñez C, Perdomo JM, Abel MD, Meineri M (2022). Preintervention imaging and intraoperative management care of the hypertrophic obstructive cardiomyopathy patient. Asian Cardiovasc Thorac Ann.

